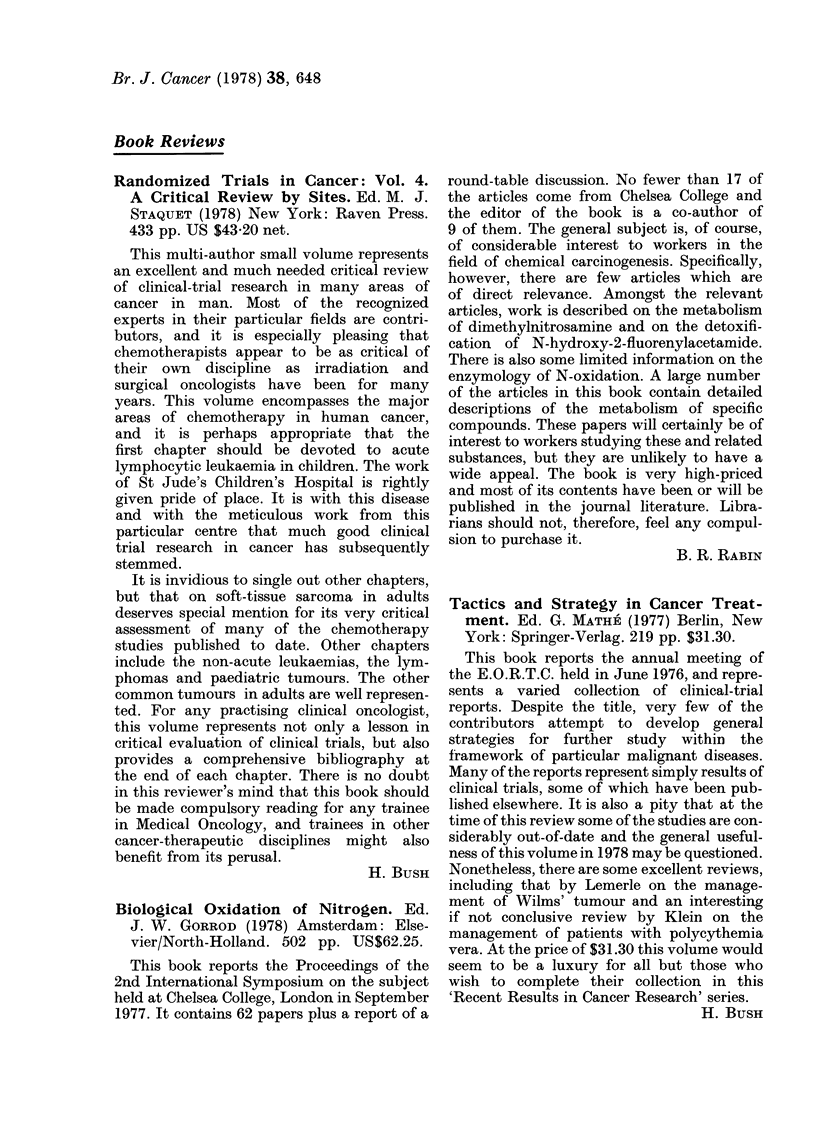# Tactics and Strategy in Cancer Treatment

**Published:** 1978-11

**Authors:** H. Bush


**Tactics and Strategy in Cancer Treatment.** Ed. G. MATHÉ (1977) Berlin, New York: Springer-Verlag. 219 pp. $31.30.

This book reports the annual meeting of the E.O.R.T.C. held in June 1976, and represents a varied collection of clinical-trial reports. Despite the title, very few of the contributors attempt to develop general strategies for further study within the framework of particular malignant diseases. Many of the reports represent simply results of clinical trials, some of which have been published elsewhere. It is also a pity that at the time of this review some of the studies are considerably out-of-date and the general usefulness of this volume in 1978 may be questioned. Nonetheless, there are some excellent reviews, including that by Lemerle on the management of Wilms' tumour and an interesting if not conclusive review by Klein on the management of patients with polycythemia vera. At the price of $31.30 this volume would seem to be a luxury for all but those who wish to complete their collection in this 'Recent Results in Cancer Research' series.

H. BUSH